# Research on the thermal performance of wall insulation materials and their impact on energy consumption in nearly zero-energy buildings

**DOI:** 10.1371/journal.pone.0338544

**Published:** 2026-01-28

**Authors:** Jun Xu, Yukun Zhu, Yu Gao, Rongshui Qin

**Affiliations:** 1 Chizhou University, Chizhou, People’s Republic of China; 2 University of Science and Technology of China, Hefei, People’s Republic of China; 3 School of Environment and Energy Engineering, Anhui Jianzhu University, Hefei, People’s Republic of China; 4 School of Architectural and Art, HeFei City College, Hefei, People’s Republic of China; Central Queensland University, AUSTRALIA

## Abstract

This study explores the thermal performance of wall insulation materials and their impact on the energy consumption of nearly zero energy buildings (NZEBs) in the hot summer and cold winter climate zone of central and southern Anhui Province, China. A bidirectional environmental chamber was constructed at university to evaluate two graphite-enhanced insulation materials—graphite extruded polystyrene (XPS, extruded polystyrene board; GXPS, graphite extruded polystyrene board) and graphite expanded polystyrene (SEPS, graphite molded polystyrene board)—under simulated seasonal temperature and humidity conditions. Experimental results show that GXPS exhibits superior thermal insulation performance during winter and transitional seasons, with a thermal conductivity as low as 0.023 W/(m⋅K) and stable surface heat flux under large temperature differentials. In contrast, SEPS performs notably well in hot and humid summer conditions, featuring uniform internal temperature distribution and pronounced thermal inertia, which effectively delays heat transfer. Dynamic thermal response analysis reveals that GXPS has a fast cooling response suited for winter, while SEPS demonstrates delayed heating behavior that mitigates summer heat stress. Building energy simulation results indicate that a GXPS insulation thickness of 50 mm achieves optimal energy savings in the region, with annual energy reduction rates between 13.6% and 22.0%. The complementary thermal properties of GXPS and SEPS provide a promising envelope design strategy for regional NZEBs, contributing to energy efficiency improvement and supporting China’s carbon neutrality targets.

## 1 Introduction

Climate change and the ongoing global energy crisis are driving significant changes in the built environment, with buildings accounting for approximately 40% of global final energy consumption and a substantial share of greenhouse gas emissions [1–4]. To address these challenges, developed regions such as the European Union and the United States have established advanced strategies for the implementation of nearly zero energy buildings (NZEBs), setting increasingly stringent requirements on building envelope performance, energy efficiency, and the use of renewable technologies [5–7]. In China, after achieving a three-step building energy efficiency roadmap (30–65% improvements), the strategic focus has shifted toward the promotion of ultra-low energy and NZEB standards as part of the country’s carbon neutrality commitments [8].

The building envelope plays a crucial role in moderating indoor temperatures and reducing energy demand. Recent studies have shown that optimizing insulation materials [9,10], window systems, and shading strategies can substantially improve envelope performance, particularly when tailored to distinct climatic regions [11–15]. Experimental and simulation-based research demonstrates that thermal performance and moisture resistance requirements for building envelopes vary markedly across different zones, necessitating climate-specific solutions [16–18].

Researchers have employed various advanced methods to enhance these designs. For instance, multi-objective optimization (MOO) and BIM-based approaches have been widely applied to balance energy and cost [19–22], while specific strategies for rural and public buildings have been developed to address local challenges [23–25]. Strategies such as building integrated photovoltaics (BIPV) [26]. and optimized Indigenous material usage [27,28]. have also been explored to address energy demands.

Central and southern Anhui province presents a classic case of a hot summer and cold winter climate, featuring high year-round humidity, hot summers, and cold winters. This duality imposes significant functional requirements: building materials must provide effective heat insulation and moisture protection in summer, coupled with strong insulation and airtightness in winter. Current national standards specify exterior wall thermal transmittance between 0.15–0.4 W/m^2^·K for residential and 0.1–0.2W/m^2^·K for public buildings—standards that underscore the importance of high-performance insulation materials in this region. Conventional insulation often fails to meet these specifications without substantial thickness, especially when *λ*-values exceed 0.03 W/(m⋅K).

Recent literature has explored emerging insulation technologies including eco-friendly nanomaterials [29], bio-based or vacuum insulation composites [30–32], and phase change material (PCM) integration. The integration of PCM within insulation materials can further smooth indoor temperature fluctuations and enhance seasonal heat storage [33–37]. Efforts have also been made to mitigate thermal bridges and optimize multilayer composites (such as Trombe walls or PCM-concrete) for hot summer and cold winter climates , resulting in notable enhancements in both energy savings and occupant comfort [38–41].

In terms of practical application, graphite-enhanced polystyrene products (such as SEPS and GXPS) are widely adopted for their low thermal conductivity and fire safety benefits [42–44]. These materials have shown improved dynamic thermal response and adaptability to variable climatic conditions, making them suitable candidates for NZEB envelopes in challenging climates [45–47]. Nevertheless, there remain unresolved research questions regarding their long-term performance, dynamic response under fluctuating seasonal conditions, and the optimization of insulation thickness for maximum energy efficiency. Further studies are warranted to clarify the dynamic performance of advanced insulation systems in hot summer and cold winter contexts and to establish data-driven guidelines for regional NZEB envelope design. The present work addresses these gaps through combined experimental investigation and simulation analysis of graphite-enhanced insulation materials under realistic climatic scenarios in central and southern Anhui, aiming to underpin technical solutions that support both energy efficiency and China’s carbon neutrality targets.

## 2 Heat transfer model of building envelope

**Fig 1:**
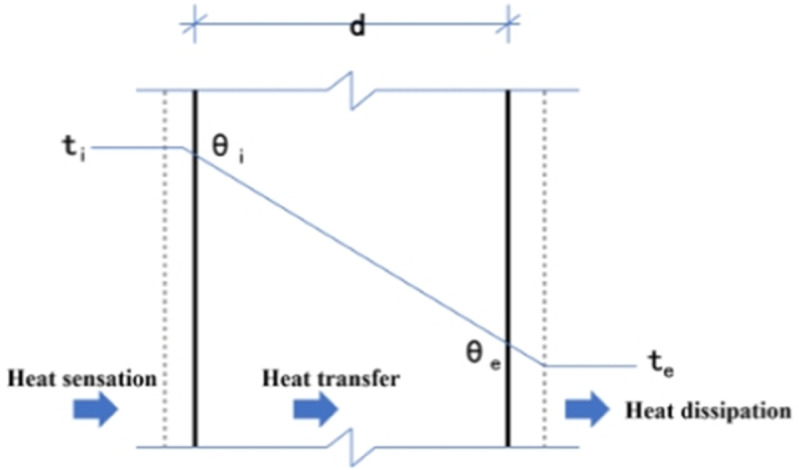
Schematic diagram.

Building envelopes are categorized into transparent and opaque structures. Opaque structures do not allow light penetration and primarily refer to solid walls and roofs; transparent structures mainly include windows and glass curtain walls. Heat exchange between the envelope and external environment is typically represented as heat gains, which can be divided into sensible heat gains and latent heat gains. Latent heat is closely related to water vapor partial pressure in the air, usually expressed as moisture content, while heat gain generally refers to sensible heat gain. The heat transfer mechanisms differ between transparent and opaque structures: solar radiation gain primarily occurs through transparent structures, while conduction is the main heat transfer mechanism for opaque structures. This research primarily focuses on analyzing the thermal performance of insulation materials for opaque structures and their impact on building energy consumption. The heat transfer in opaque building envelopes mainly involves solar radiation gain on the external surface and conduction through the wall. Heat transfer in building envelopes includes three basic processes: surface sensible heat exchange, component heat transfer, and surface radiation, with the main heat transfer methods for each process shown in Table 1. The schematic diagram is shown in Fig 1.

**Table 1:** Basic processes of heat transfer in building envelope structures and their primary modes of heat transfer.

Process name	Primary modes of heat transfer
**Surface sensible heat process Component heat transfer model Surface heat dissipation process**	Convection Radiation Conduction Convection Radiation

**Fig 2:**
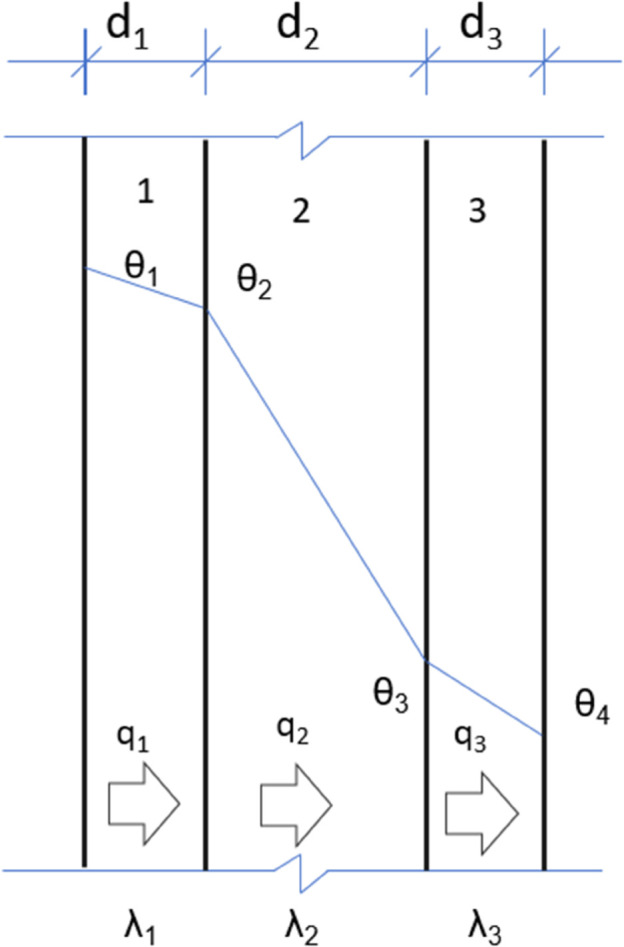
Heat transfer of material layers in opaque envelope.

According to the steady-state heat conduction equation, the conduction heat per unit time and unit area through each material layer of an opaque building envelope (wall) (as shown in Fig 2) is:

$$q_{1}=\frac{\lambda_{1}}{d_{1}}(\theta_{1}-\theta_{2}),\;q_{2}=\frac{\lambda_{2}}{d_{2}}(\theta_{2}-\theta_{3})\;q_{3}=\frac{\lambda_{3}}{d_{3}}(\theta_{3}-\theta_{4}),\;q_{n}=\frac{\lambda_{n}}{d_{n}}(\theta_{n}-\theta_{n+1})$$ (1)

In Eq (1): - *q*_1_, *q*_2_, *q*_3_: Heat transfer per unit time and per unit area through each material layer (heat flux density), W/m^2^; - *θ*_1_, *θ*_2_, $\theta_{3}$, $\theta_{4}$: Surface temperatures of each material layer, °C; - $\lambda_{1}$, $\lambda_{2}$, $\lambda_{3}$: Thermal conductivity of each material layer, W/(m⋅K); - *d*_1_, *d*_2_, *d*_3_: Thickness of each material layer, m.

## 3 Experimental design for thermal performance of typical insulation materials

### 3.1 Experimental chamber construction

**Fig 3:**
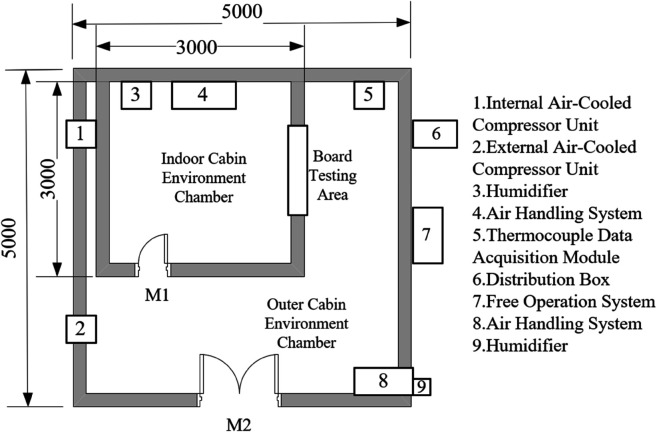
Plan of bidirectional cabin at Anhui Jianzhu University.

To accurately reflect the performance of insulation materials under the climate conditions of central and southern Anhui, this study employed a bidirectional experimental chamber for testing (floor layout shown in Fig 3). The chamber was constructed using prefabricated insulation panels, with both internal and external environment chambers equipped with multifunctional comprehensive environmental treatment systems, adopting an airflow organization form of top perforated plate air supply and bottom return air. The air treatment system includes refrigeration and dehumidification equipment, heating equipment, pneumatic distribution systems, and humidification equipment, with temperature and humidity precisely controlled through coarse adjustment by refrigeration and dehumidification equipment and fine adjustment by heating and humidification equipment. Each chamber is equipped with an air-cooled compression condensation system, with the compressor adopting a multi-head design that can be independently controlled to achieve graded cooling capacity adjustment. The temperature and humidity control range of the experimental chamber: external chamber –15°C, internal chamber 35°C, temperature control accuracy ±0.5°C, relative humidity 30% 95%, accuracy ±5%. Two walls of the internal chamber overlap with the external chamber, while the other two are light-gauge steel stud structures. The internal chamber has a 1m × 1m adjustable test opening (shown in Fig 3) for installing insulation material modules to be tested. This study selected 4mm thick graphite extruded polystyrene board (GXPS) and 3mm thick graphite expanded polystyrene board (SEPS) as test subjects, both with dimensions of 1.2m × 1.2m, collecting data through heat flow meters and thermocouples to analyze their thermal performance. The physical property parameters of GXPS and SEPS insulation boards are shown in the Table 2.

**Table 2:** Physical property parameters table of GXPS and SEPS thermal insulation boards.

Parameter	GXPS	SEPS
**Thermal conductivity**	0.028–0.035 W/(m⋅K)	0.033–0.040 W/(m⋅K)
**Density**	28–45 kg/m^3^	15–30 kg/m^3^
**Compressive strength**	150–700 kPa	70–200 kPa
**Water absorption**	*leq*1.0%	≤2.0%
**Temperature coefficient**	0.4%–0.7%/°C	0.5%–0.8%/°C

### 3.2 Experimental condition settings

The experimental temperature and humidity conditions were comprehensively determined based on the climate characteristics of central and southern Anhui (using Hefei and Huangshan as examples), building energy demands, standard specifications, and thermodynamic principles. The main climate parameters of Hefei and Huangshan are shown in the Table 3. Central and southern Anhui has a hot summer and cold winter climate, with high temperatures in summer increasing air conditioning energy consumption and low temperatures in winter enhancing heating demands, requiring building envelopes to consider both insulation and heat isolation. Based on this, the internal chamber temperature was set at two levels: 22°C and 26°C, while the external chamber temperature was divided into three levels (3–5°C, 18–22°C, 28°C) for measurement. Considering the region’s year-round high humidity characteristics, the internal chamber humidity was maintained at 50%, while the external chamber humidity was adjusted as needed between 50% and 90%. Condition A involved continuous testing with the external chamber at normal temperature and the internal chamber at normal temperature, with specific parameters shown in Table 4.

**Table 3:** Climatic characteristic parameter table for typical South-Central Anhui Cities.

Climate Parameter	Hefei City	Huangshan City
**Annual average temperature**	16.0°C	15.5°C
**Summer average high temperature**	32–38°C	28–32°C
**Winter average low temperature**	–5–0°C	–8–2°C
**Annual average relative humidity**	75%–80%	80%–85%
**Climate Parameter**	Hefei City	Huangshan City

**Table 4:** Bidirectional chamber experimental conditions table.

Working conditions	Internal Chamber Temperature Setting (°C)	Internal Chamber Humidity Setting (%)	External Chamber Temperature Setting (°C)	External Chamber Humidity Setting (%)	Insulation Material Type/ Thickness (mm)	Base Layer Thickness (mm)	Duration (h)
**A0**	26	50	3	80	GXPS/40	25	24
**A**	26	50	18	75	GXPS/40	25	24
**B1**	22	50	5	60–80	GXPS/40	20	6
**B2**	26	50	22	50–90	GXPS/40	20	6
**B3**	26	50	28	75–90	GXPS/40	20	6
**C1**	22	50	5	60–80	SEPS/30	20	6
**C2**	26	50	22	50–90	SEPS/30	20	6
**C3**	26	50	28	75–90	SEPS/30	20	6

### 3.3 Measurement point layout

The experimental equipment is shown in Table 5, with three sets of conditions (A/B/C) established. Among them:

**Table 5:** Experimental equipment selected.

Instrument Name	Instrument Model	Instrument Range
**Multi-Channel Temperature and Heat Flux Tester**	JTNT-A	02000W/m^2^ –50°C–120°C
**Thermocouple**	OMEGA-K	–200–1250°C

⋅ Condition A has 13 temperature measurement points and 2 heat flux density measurement points (3 on external surface, 5 in middle layer, 5 on internal surface)

⋅ Conditions B/C each have 15 temperature measurement points and 6 heat flux density measurement points (5 each on external/middle/internal surfaces)

The distribution of measurement points is shown in Figs 4–7 and Figs 8–13(a,b,c), where rectangular markers denote temperature measurement points and circular markers represent heat flux density measurement points.

**Fig 4:**
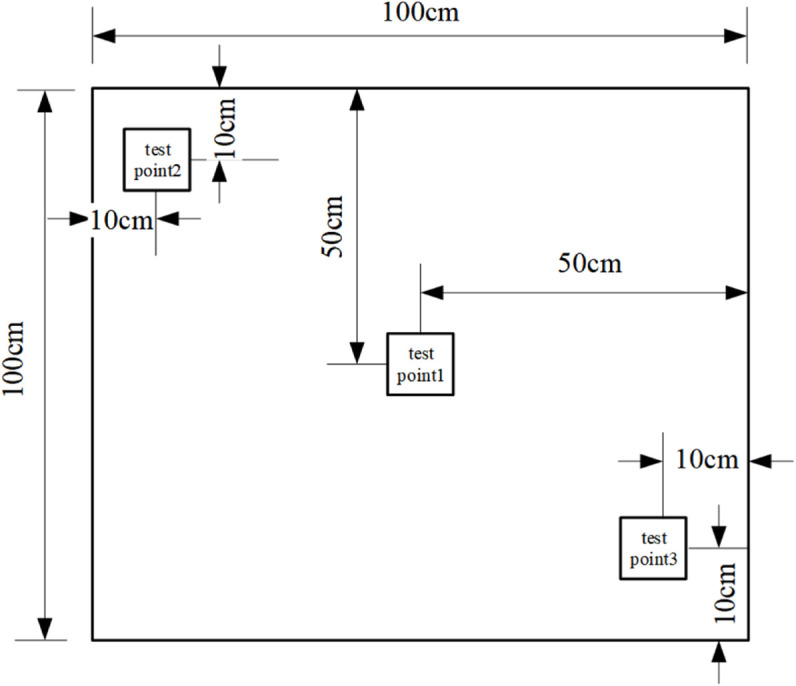
Layout of points on external foundation wall.

**Fig 5:**
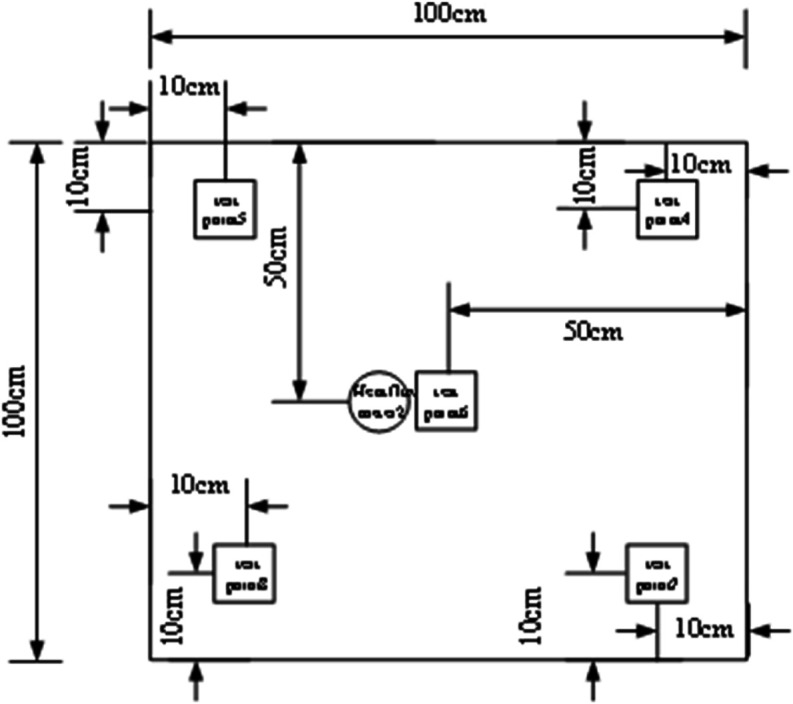
Layout of points between panel and foundation wall.

**Fig 6:**
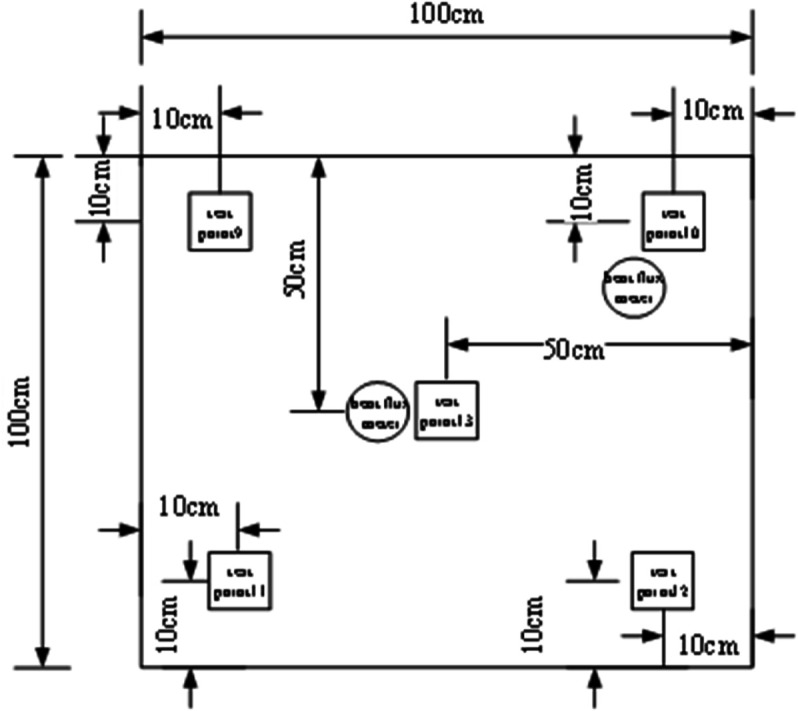
Layout of points on internal GXPS board.

**Fig 7:**
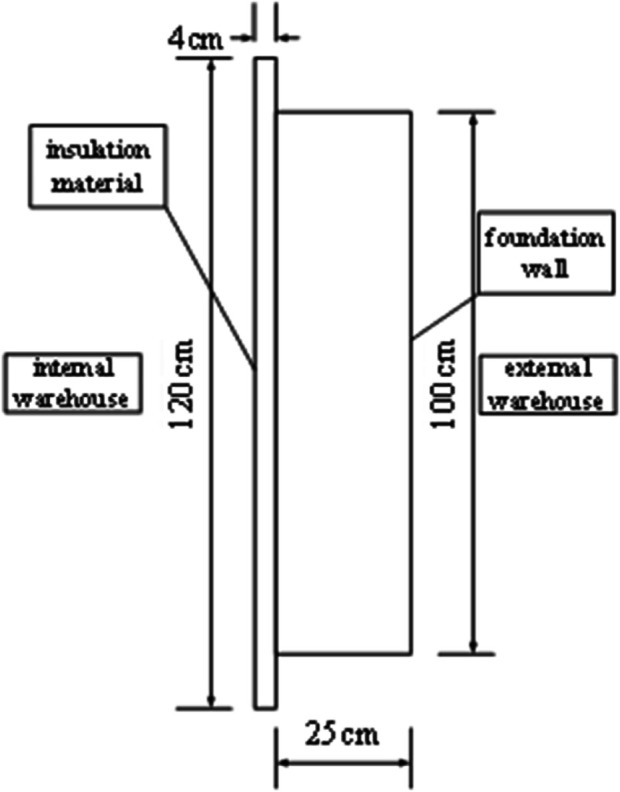
Layout of points on internal GXPS board.

**Fig 8:**
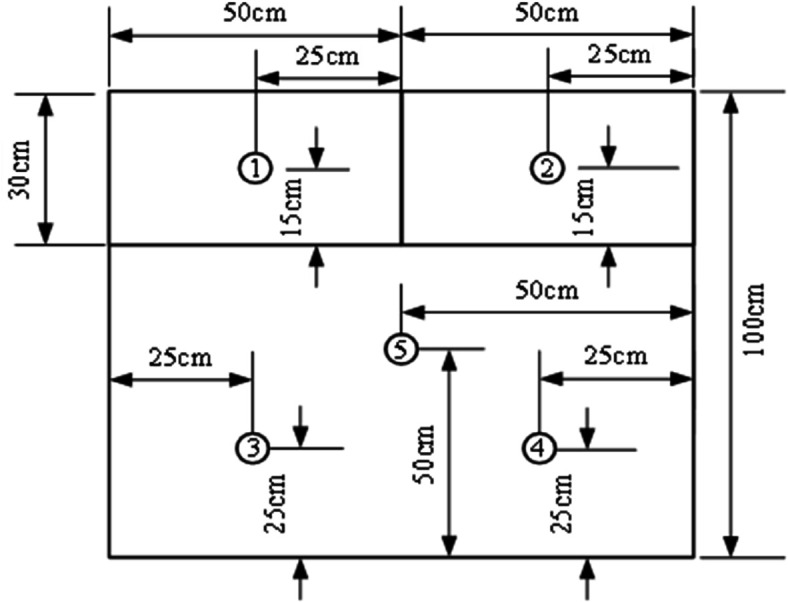
Layout of points on outer surface of external concrete warehouse.

**Fig 9:**
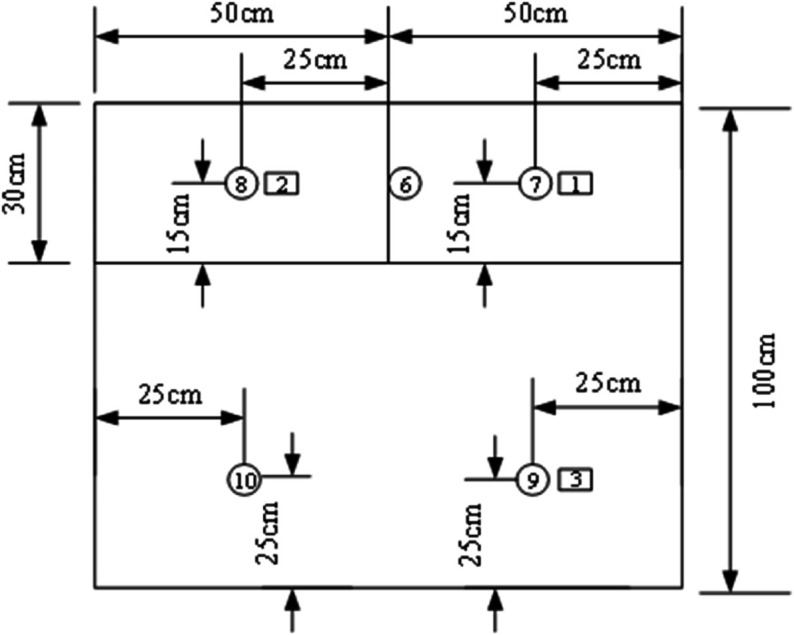
Layout of points between slab and foundation wall.

**Fig 10:**
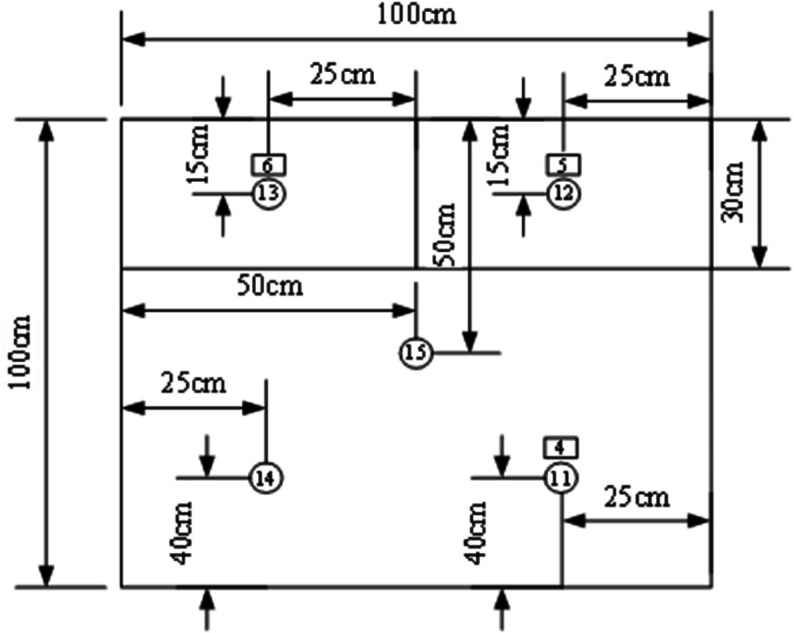
Layout of points on internal warehouse slab.

**Fig 11:**
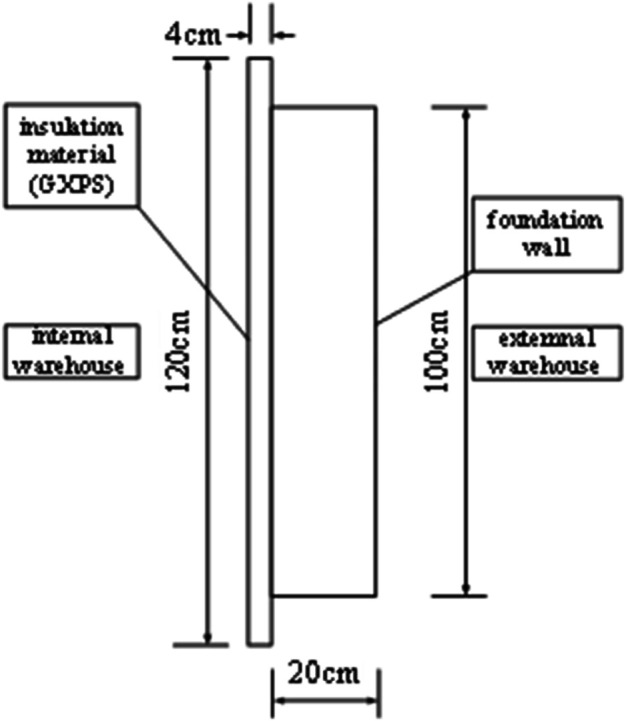
Condition B elevation drawing.

**Fig 12:**
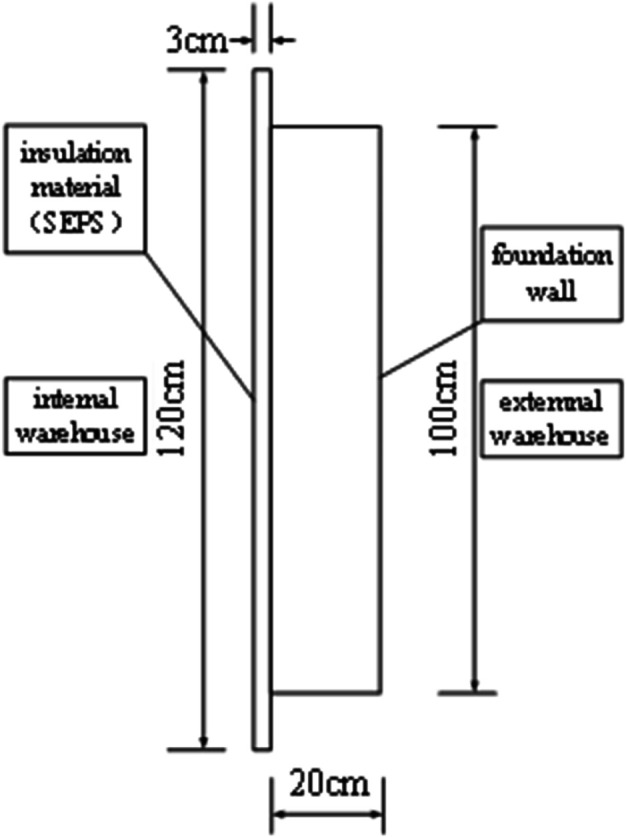
Condition C elevation drawing.

**Fig 13:**
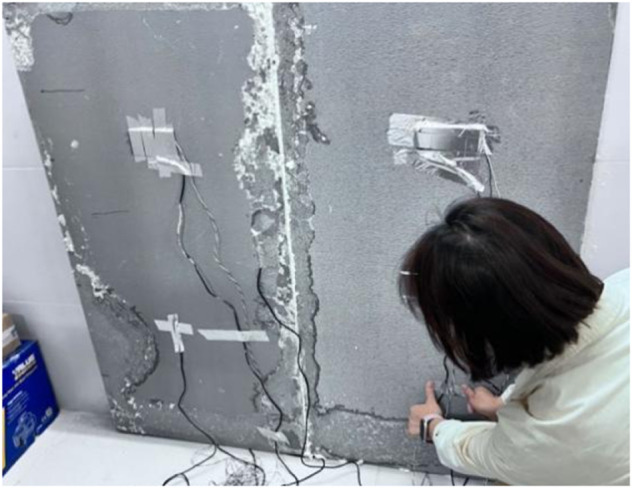
On-site measurement point layout.

### 3.4 Experimental procedure

The performance of building envelopes has a decisive impact on building energy consumption. To study the performance of insulation materials in the climate of central and southern Anhui, experiments were conducted in the bidirectional chamber at Anhui Jianzhu University, focusing on analyzing the thermal performance of two new types of insulation materials, GXPS and SEPS, collecting data on temperature and humidity inside and outside the chamber, as well as temperature and heat flux density at various measurement points of the panels.

The experiment was divided into three stages:

⋅ Condition A: Baseline testing without insulation materials

⋅ Conditions B/C: Testing the performance of GXPS and SEPS respectively in transitional seasons, summer, and winter

During the testing process, infrared imaging monitoring was conducted on the corresponding measurement areas and various measurement points inside and outside the chambers at half-hour intervals to assist in data assessment.

The panels were fixed using an adhesive method (dry construction), which can avoid thermal bridge effects compared to mechanical fixation. Specific steps:

⋅ Before testing Condition A, thermal conductive silicone grease was applied to the interfaces of multi-channel temperature/heat flow measuring instruments and thermocouple contacts to ensure stable contact;

⋅ The chamber temperature was adjusted through the central control system, and data was collected after the temperature field stabilized;

⋅ When testing Conditions B/C, insulation panels were fixed with special adhesives using the dry bonding method, and continuous monitoring was carried out following the same procedure.

This scheme, through strict control of boundary conditions and synchronous collection of multiple parameters, can accurately quantify the patterns of thermal resistance change in the two materials under different seasonal conditions, providing empirical data support for building envelope design in hot summer and cold winter regions.

### 3.5 Experimental calibration

Temperature sensor (thermocouple) calibration:

A standard constant temperature bath (accuracy ±0.1°C) was used for multi-point calibration within the experimental temperature range (–15°C-50°C) to verify its linearity and consistency with the ±0.5°C accuracy requirement. Calibration points selected: –10°C, 0°C, 20°C, 35°C, 50°C.

Heat flow meter calibration:

Standard reference panels with known thermal conductivity were used for calibration under steady-state heat flow conditions to ensure heat flux density measurement error *leq*3%.

Multi-channel data acquisition system synchronization verification:

All channels were connected to the same standard signal source (such as a constant current/voltage generator) to detect the time synchronization and signal response consistency between channels, eliminating delay differences between channels.

## 4 Analysis of thermal performance experiments on insulation materials

### 4.1 Experimental calibration

**Fig 14:**
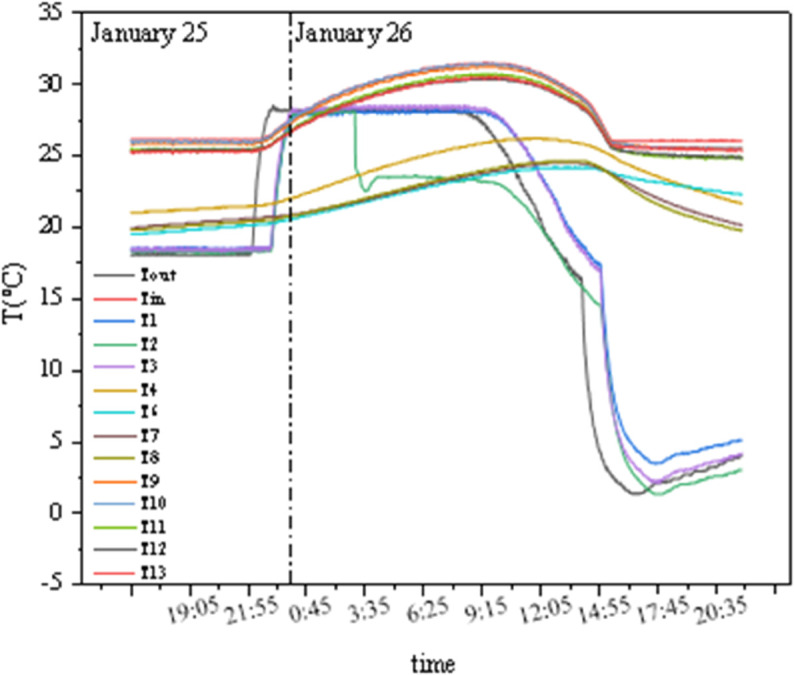
Temperatures of points in environmental warehouse (A).

**Fig 15:**
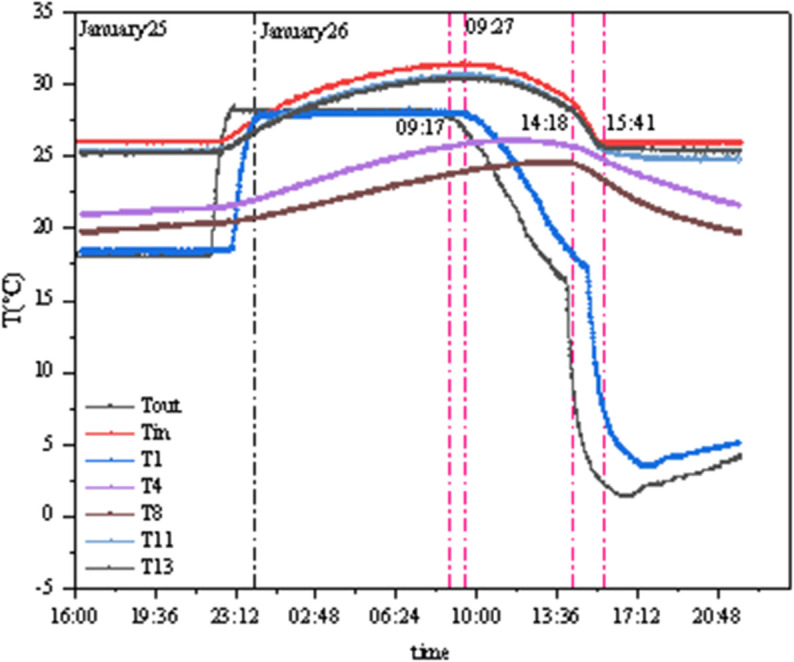
Instant thermal response of three interfaces (A).

As shown in Figs 14 and 15, T1 and T3 represent the external chamber surface temperatures, T4–T8 represent the middle layer temperatures, and T9–T13 represent the internal chamber surface temperatures. Before the experiment started, the temperature and humidity of the internal and external chambers reached the set values, and testing was initiated after the internal and external surface temperatures stabilized. As seen in Fig 14, the concrete panel temperature responds rapidly to changes in internal and external chamber temperatures, while the insulation properties of the insulation materials remain stable. The temperature difference between the two sides of the GXPS board ranges from a minimum of 1.655°C to a maximum of 7.125°C, with an average of 5.195°C, while the maximum temperature difference between the internal and external chambers reaches 25°C. In Fig 15, Tout represents the external chamber temperature, and Tin represents the internal chamber temperature. When the indoor environment temperature rises, the aerated concrete surface responds rapidly to changes in the external chamber temperature, while the internal surface temperature of the GXPS board responds more slowly to heat; conversely, when the indoor environment temperature decreases, the internal and external surface temperatures of the specimen respond rapidly to changes in the internal chamber temperature, with noticeable changes occurring within 10 minutes, indicating that the thermal response of insulation materials during environment warming is slower than during environment cooling.

### 4.2 Conditions B1 and C1

**Fig 16:**
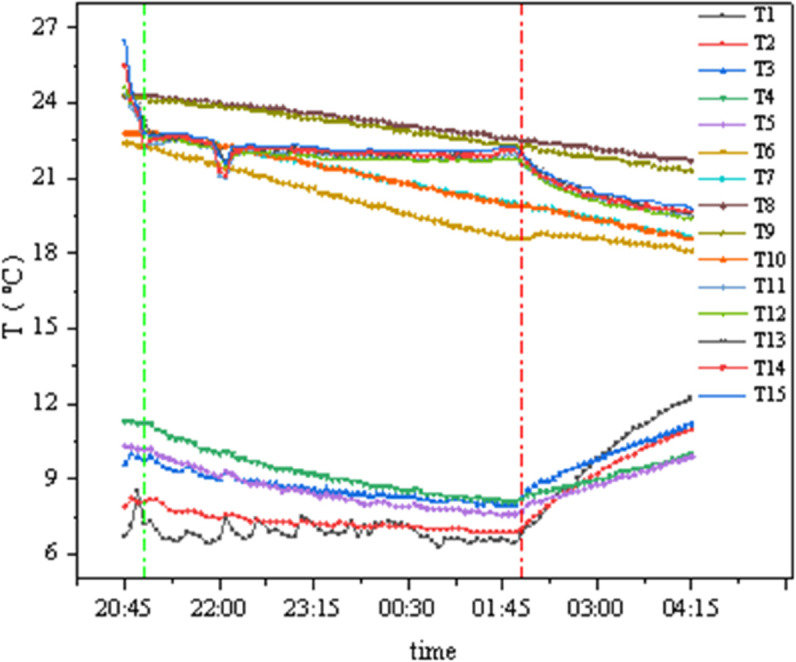
Temperature curves of GXPS (B1C1).

**Fig 17:**
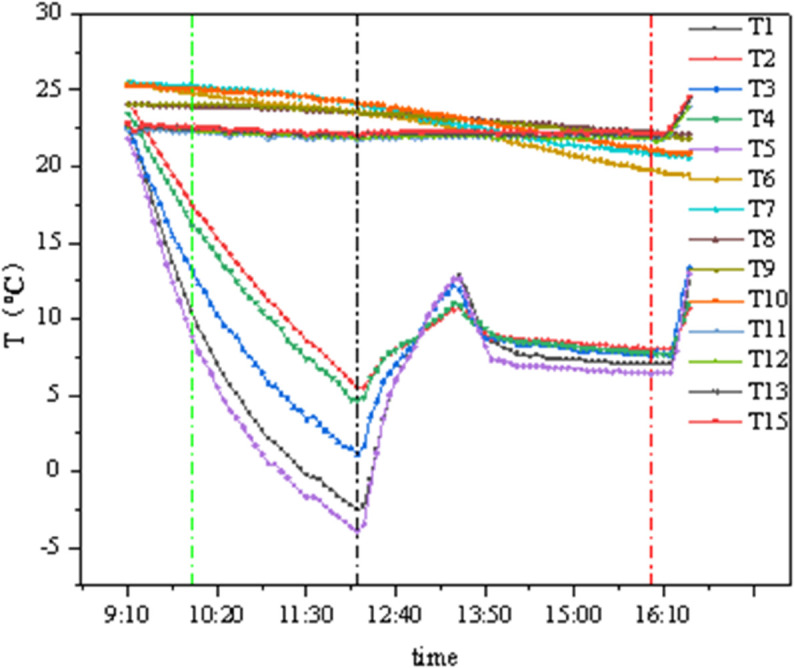
Temperature curves of SEPS (B1C1).

As shown in Figs 16 and 17, the internal chamber temperature is set at 22°C with 50% humidity, and the external chamber temperature is 5°C with 60%–80% humidity. The green reference line indicates the moment when the external chamber temperature reached its set stable value, and the red reference line indicates the moment when the system was shut down. T1–T5 represent the concrete external surface temperatures, T6–T10 represent the insulation material surface temperatures, and T11–T15 represent the concrete internal surface temperatures. In Fig 16, data collection began when the external wall surface temperature was constant, while in Fig 17, testing started when the external temperature reached its set value.

From the figure, it can be observed that the temperature fluctuation on the internal surface of the insulation material is extremely minimal, while the external surface temperature gradually decreases with the external chamber temperature, albeit at a slow rate. Based on the average values of various measurement points, calculations show: the GXPS board has a minimum temperature difference of 0.05°C and a maximum of 1.93°C; the SEPS board has a minimum temperature difference of 0.685°C and a maximum of 2.355°C.

The framework of this empirical study integrates three theoretical perspectives: (1) Modified UTAUT2 constructs as the core behavioral determinants framework; (2) Ethnic identity as the external influence mechanism while employing EIS as the measurement; and (3) a modified AISAS model that conceptualizes user behavior patterns. Fig 3 is the conceptual road map of integrated model.

### 4.3 Conditions B2 and C2

**Fig 18:**
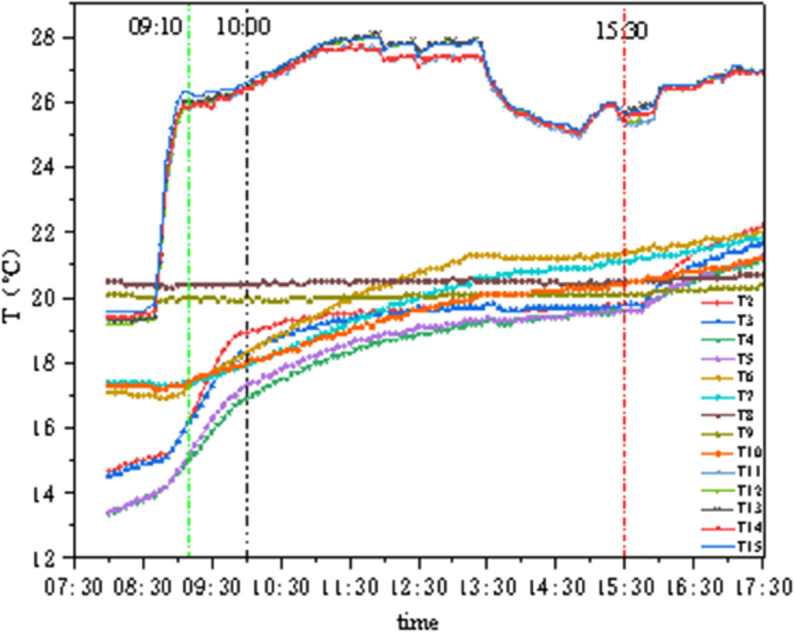
Temperature variation of GXPS (B2,C2).

**Fig 19:**
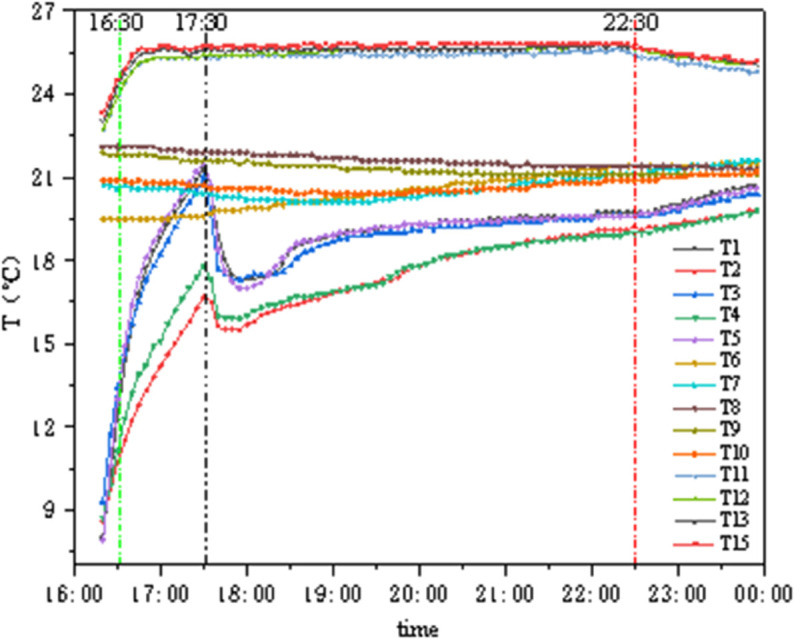
Temperature variation of SEPS (B2,C2).

As shown in Fig 18, under these two conditions, the internal chamber temperature is 26°C with 50% humidity, and the external chamber temperature is 20°C with 50%–90% humidity. The green line indicates the moment when internal and external temperatures reached their set values, and the black line indicates the moment when the concrete external surface reached a stable value. In Fig 18, the internal surface of the GXPS insulation material responds rapidly to the internal chamber temperature rise, with a fast warming rate; however, after the system is shut down, the external surface of the panel warms up rapidly, while the insulation material still maintains relative stability. In Fig 19 with SEPS insulation material, the concrete external surface temperature rises rapidly with the external temperature, the middle measurement points T6–T10 temperatures remain basically constant, and the internal surface temperature rapidly rises to the internal chamber temperature and then remains stable. Calculations show that the SEPS board has a minimum temperature difference of 3.17°C and a maximum of 4.865°C, while the GXPS board has a temperature difference of approximately 6.8°C. It is evident that both materials exhibit good thermal stability in maintaining indoor temperature.

### 4.4 Conditions B3 and C3

**Fig 20:**
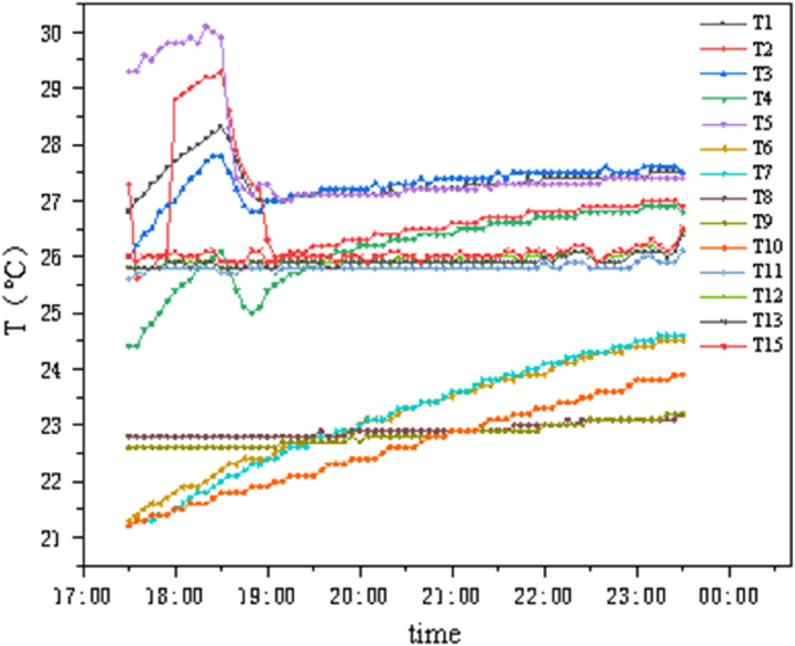
Temperature variation of GXPS (B3,C3).

**Fig 21:**
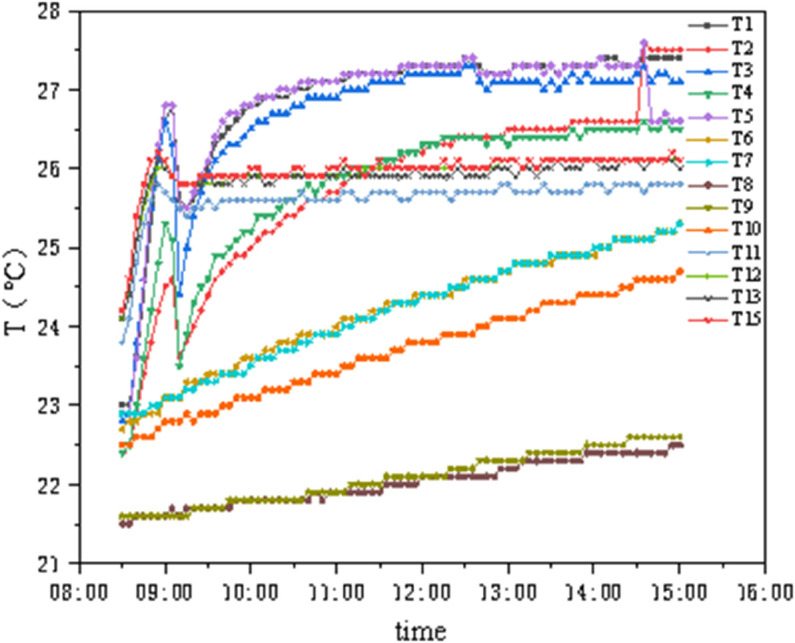
Temperature variation of SEPS (B3,C3).

As shown in Figs 20 and 21, under these two conditions, the internal chamber temperature is 26°C with 50% humidity, and the external chamber temperature is 28°C with 75%–90% humidity. The temperature change amplitude on the internal and external surfaces of the insulation materials is extremely small: the GXPS board has an internal-external surface temperature difference of approximately 3°C, with a minimum of 2.185°C and a maximum of 3.98°C; the SEPS board has a minimum temperature difference of 0.39°C and a maximum of 3.665°C, indicating that both materials can maintain stable thermal performance under the specified conditions.

### 4.5 Analysis of heat flux density and thermal conductivity

Based on the aforementioned heat flux density, surface temperature, and changes in internal and external chamber temperatures, Eq 3.4 is used to calculate steady-state heat transfer through a flat wall, treating the fixed set temperatures of the internal and external chambers as steady-state conditions. The average heat flux density is determined through the average temperature of measurement points, and then the thermal conductivity is calculated, with results shown in Tables 6 and 7. The SEPS board has a more uniform temperature distribution, with a thermal conductivity of approximately 0.03 W/(m⋅K); the GXPS board achieves a thermal conductivity of 0.023 W/(m⋅K) during the transitional season, showing more significant insulation effects.

**Table 6:** Thermal conductivity of GXPS board under various operating conditions.

Operating Conditions	Outer Surface Temperature01(^∘^C)	Inner Surface Temperature02(^∘^C)	q W/m2	λ W/(m⋅K)
**B1**	21.97	22.07	–0.53	0.036
**B2**	19.98	26.76	3.94	0.023
**B3**	22.94	25.93	1.78	0.024

**Table 7:** Thermal conductivity of SEPS board under various operating conditions.

Operating Conditions	Outer Surface Temperature01(^∘^C)	Inner Surface Temperature02(^∘^C)	q W/m2	λ W/(m⋅K)
**C1**	23.13	22.07	–0.72	0.027
**C2**	20.90	25.54	4.11	0.027
**C3**	23.35	25.85	2.46	0.031

**Fig 22:**
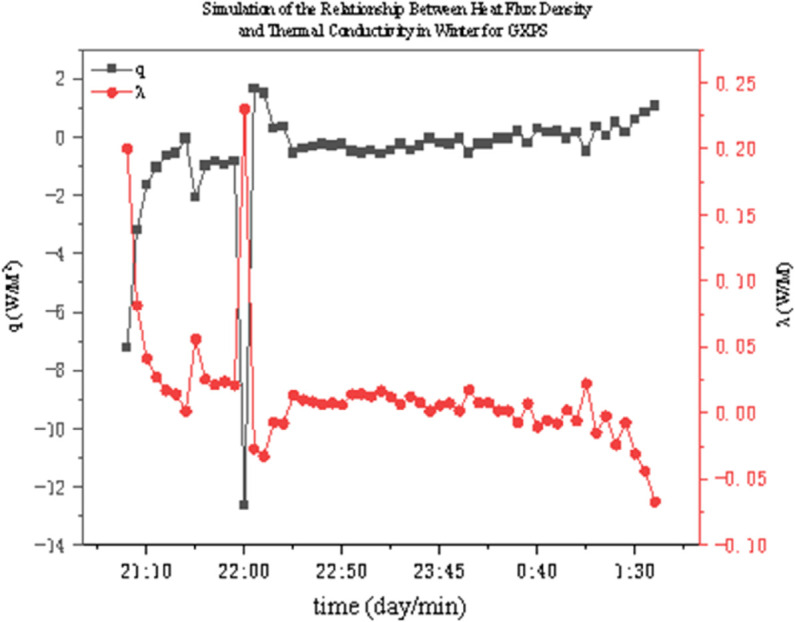
GXPS Heat flux vs conductivity (B1).

**Fig 23:**
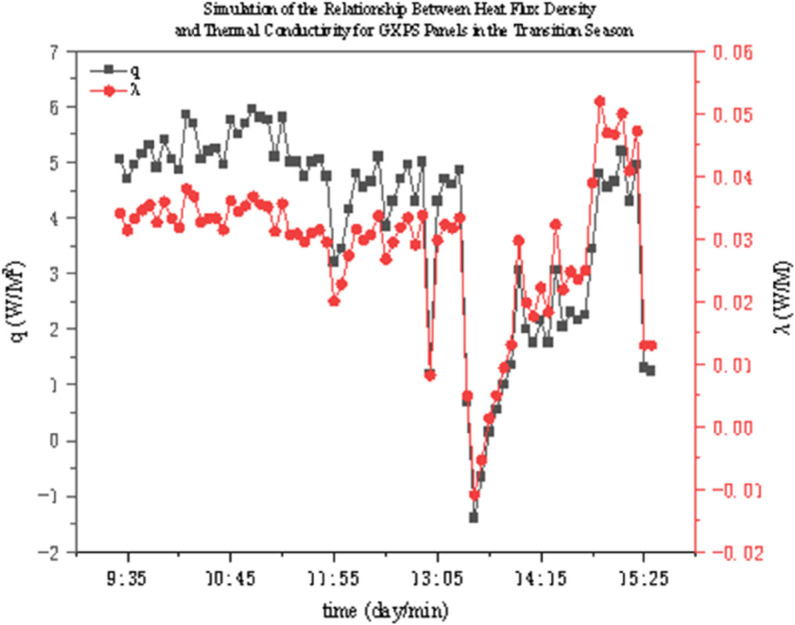
GXPS Heat flux vs conductivity (B2).

**Fig 24:**
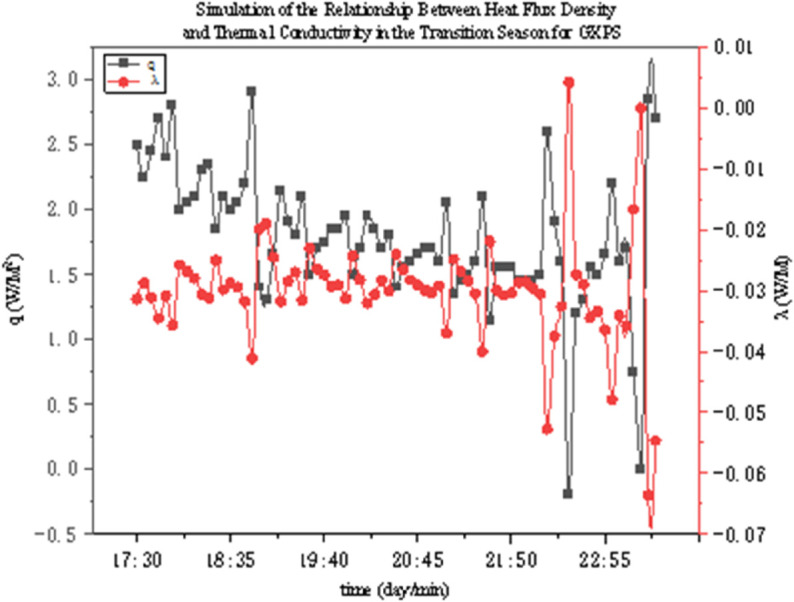
GXPS Heat flux vs conductivity (B3).

**Fig 25:**
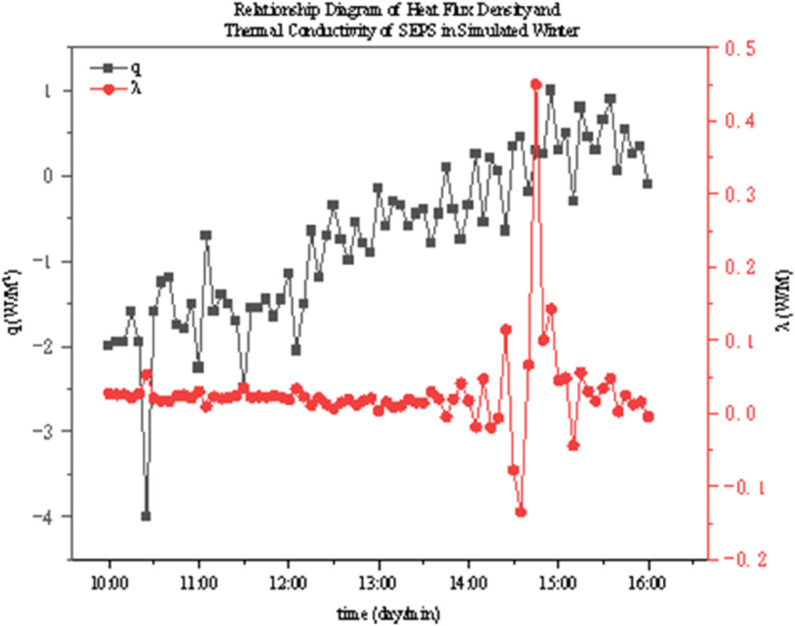
SEPS Heat flux vs conductivity (C1).

**Fig 26:**
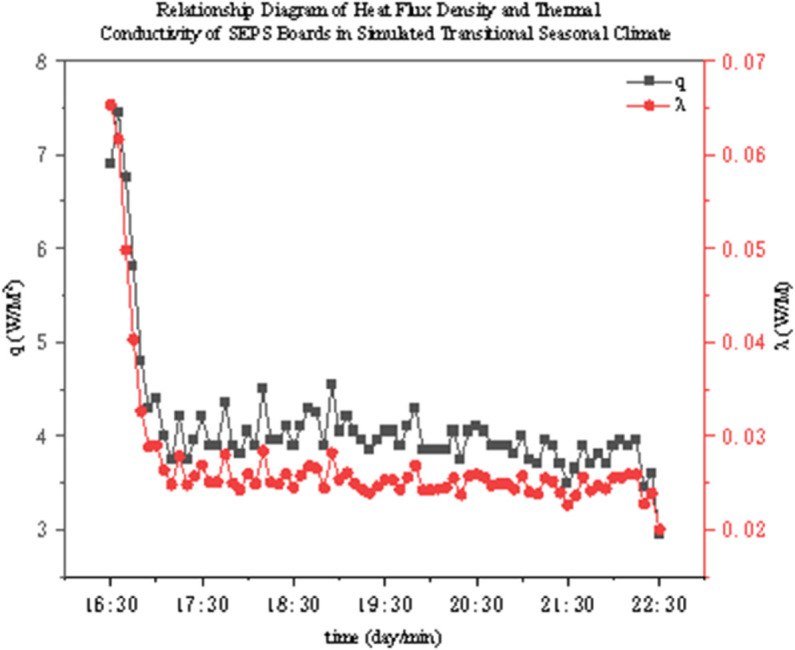
SEPS Heat flux vs conductivity (C2).

**Fig 27:**
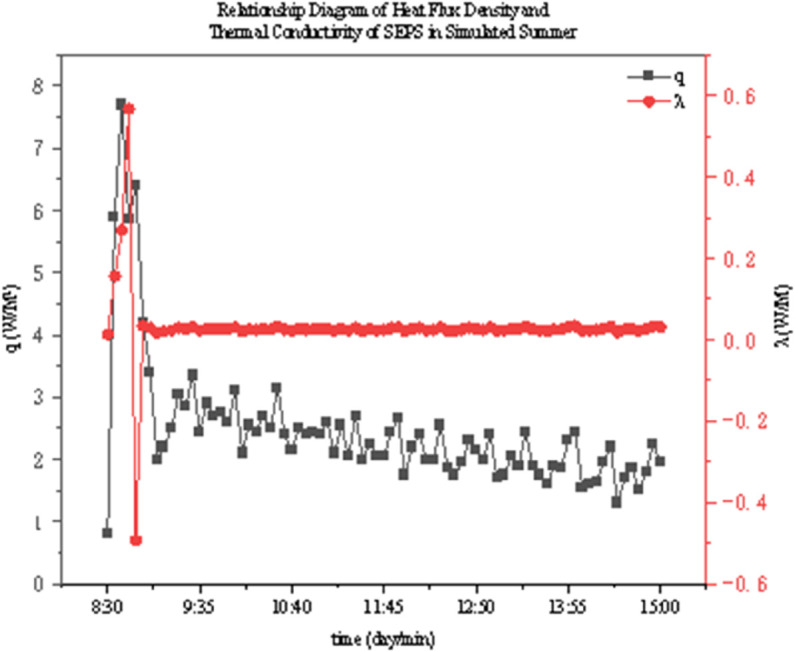
SEPS Heat flux vs conductivity (C3).

The thermal conductivity of two thermal insulation materials under different operating conditions was determined through experiments. As shown in Figs 22–27, it presents the relationship curves between heat flux density and thermal conductivity of two different thermal insulation materials (GXPS board and SEPS board) under different operating conditions, where q represents heat flux density and *λ* represents thermal conductivity. The fluctuation range of thermal conductivity proves their superiority in high-efficiency thermal insulation. For example, as indicated in condition B1, after the indoor environment stabilizes, the thermal conductivity of GXPS fluctuates around 0.025 W/(m⋅K). There is an oscillation point under condition B2, which is due to the process of the internal and external temperature difference changing from internal heat and external cold to internal cold and external heat (similar to the transition season). Condition B3 shows that its thermal insulation effect is significant under the environment of external cold and internal heat (similar to winter).

The SEPS board is relatively stable, but there is an oscillation point under condition C1, which occurs when the external environment changes from a low temperature of 5°C to a normal temperature of 26°C, leading to a large fluctuation in the internal and external temperature difference. Its thermal conductivity basically oscillates around 0.03 W/(m⋅K). It can be seen that both thermal insulation materials have good thermal insulation performance, especially when the temperature difference between indoor and outdoor is large (such as winter). However, when the indoor and outdoor temperatures fluctuate, the stability of the materials will decrease. Therefore, both materials can meet the requirements in winter in central Anhui, southern Anhui, and even the hot summer and cold winter regions. But in summer, when the indoor and outdoor temperature difference is unstable, priority should be given to the rational application of these two materials.

Throughout the entire experimental process, steady-state heat transfer processes were used for testing. The temperatures of the internal and external chambers were kept constant and stable, ensuring that the heat transfer processes under various experimental conditions could accurately reflect the thermal performance of the insulation materials. According to the definition of heat flux density (which is typically defined as positive in the direction from indoors to outdoors), the heat transfer direction under this operating condition is opposite to the defined positive direction; therefore, the calculated values appear as negatives. This accurately reflects the summer condition in which heat intrudes from outside to inside.

### 4.6 Dynamic thermal response analysis of insulation materials

**Fig 28:**
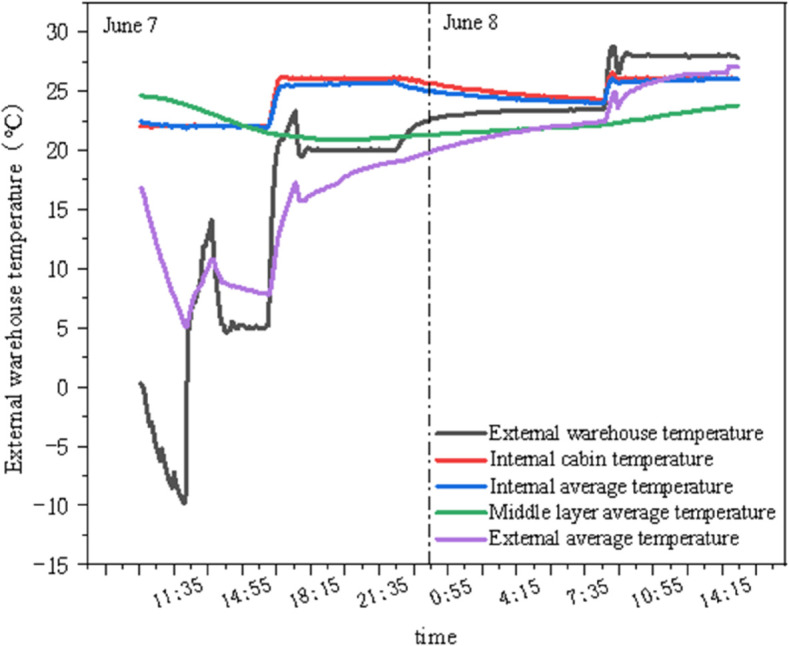
Average temperature variation of points (C1–C3).

As shown in Fig 28, Conditions C1 to C3 represent a continuous testing process where the internal chamber temperature rises from 22°C to 26°C (fluctuation ±1°C), and the external chamber temperature first drops below zero then stabilizes at 5°C, subsequently rises to approximately 20°C, and finally reaches 28°C (fluctuation ±1°C). The internal surface temperature of the insulation material consistently follows changes in the internal chamber temperature, almost unaffected by the external chamber temperature; the middle layer temperature of the wall, although jointly affected by internal and external environments, changes relatively gradually. According to the first law of thermodynamics, the intermediate temperature first gradually decreases and then slowly increases, but with a slow thermal response time. Throughout the entire experimental process, the external chamber temperature fluctuates by more than 30°C, and the maximum temperature difference between the internal and external chambers reaches 30°C, while the average temperature difference between the two sides of the insulation material consistently remains below 6.5°C, indicating its significant insulation effect and good stability.

### 4.7 Regional adaptability analysis for central and Southern Anhui

In this analysis, to evaluate the performance of the insulation materials under the most adverse humidity conditions, the upper bounds of the humidity ranges listed in Table 4 were adopted for all calculations. This choice follows the conservatism commonly used in engineering practice and aims to ensure the safety and reliability of the analytical results.

In a high-temperature and high-humidity environment (external chamber temperature 28°C, humidity 90%), the maximum temperature difference between the internal and external surfaces of SEPS is only 3.665°C, and its temperature distribution uniformity is superior to that of GXPS. The uniform temperature distribution can reduce fluctuations in heat flux density of the building envelope, indicating that it is more suitable for suppressing summer heat penetration and condensation, effectively reducing air conditioning dehumidification loads; while GXPS, due to its low thermal conductivity, shows significant insulation advantages during transitional seasons and winter, capable of reducing cooling/heating energy consumption values.

Central and southern Anhui has significant temperature differences between day and night and across seasons. Experiments show that GXPS has a fast thermal response rate during cooling processes, matching the rapid cooling climate characteristics in winter; while SEPS has higher thermal inertia during warming, which can mitigate the disturbance of high-temperature shocks during summer days on the indoor thermal environment.

Through complementary thermal characteristics (low thermal conductivity and high humidity stability), GXPS and SEPS can effectively address the climate in central and southern Anhui, providing scientific support for constructing regional nearly zero-energy building envelope systems.

### 4.8 Error analysis

(1) Systematic error of heat flow meter measurement points

In actual measurements, it is difficult for the heat transfer coefficient on the surface of the tested insulation board to achieve uniform consistency, especially in the vicinity of the heat flow meter. Therefore, the surface temperature around the heat flow meter measurement points is not uniform, resulting in a lateral heat flow on the surface of the insulation board.

During the experiment, the ideal state is to make the heat flow through the insulation board wall Q_1_ = 0, i.e., the thermal power at the heat flow meter measurement point equals the heat flowing through the wall. During the experiment, it is difficult to reduce the heat flow through the insulation board to Q_1_ = 0, so it needs to be corrected during the experiment, which can be done using Eq (2). Additionally, the precision of the sensors will produce errors in the measurement results.

In Eq (2):

$$\Phi =M\times (t_{i}-t_{e})$$ (2)

In Eq (2): – *M*: Heat flow coefficient at the insulation board heat flow meter measurement point; – *t*_*i*_: Average temperature on the inside of the insulation board; – *t*_*e*_: Average temperature on the outside of the insulation board.

(2) Boundary condition control deviation Temperature and humidity fluctuations in the environmental chamber may lead to deviations in the thermal conductivity measurements of GXPS and SEPS, with their material temperature coefficients (*a*) being:

– GXPS: a%=0.4% 0.7%/°C (i.e., for every 1°C increase in temperature, thermal conductivity increases by approximately 0.4% 0.7%);

– SEPS: a%=0.5% 0.8%/°C (i.e., for every 1°C increase in temperature, thermal conductivity increases by approximately 0.5% 0.8%).

(3) Model calculation error

The steady-state heat transfer model does not consider the contact thermal resistance caused by gaps between the concrete base panel and insulation materials. Contact thermal resistance is caused by microscopic gaps at the interface, roughness, pressure, and other factors. The experiential range of contact thermal resistance at the GXPS and SEPS interface is typically 0.01-0.05 m^2^⋅K/W, which can be corrected using Eq (3).

In Eq (3):

$$Q=\frac{\Delta T}{\sum R_{t}+R_{c}}$$ (3)

In Eq (3): – *Q*: Heat flux density at the heat flow meter measurement point, W/m^2^; – ΔT: Temperature difference between the inside and outside of the insulation board, K; – *R*_*t*_: Total thermal resistance of the system, m^2^⋅K/W; – *R*_*c*_: Contact thermal resistance, m^2^⋅K/W. Calculations show that the actual heat loss is 0.5% 2.6% less than the theoretical value. (Slightly affected by surface roughness and installation pressure)

(4) Sensor accuracy error

In experimental measurements, infrared imaging monitoring was conducted at half-hour intervals, potentially missing short-term temperature fluctuation data.

Experiments show that even under conditions with a 30°C temperature difference between internal and external chambers, the average temperature difference between the two sides of the insulation material consistently remained stable within 6.5°C, proving that the above errors have limited impact on the core conclusions.

## 5 Model establishment and operating condition setting

DeST software was selected for the building energy consumption simulation analysis in this study. DeST is applied in fields such as building energy-efficient design, HVAC system optimization, and building energy analysis. Developed to meet China’s needs for building energy conservation and environmental optimization, it integrates theories from building physics, heat transfer, and fluid mechanics, enabling accurate simulation of building energy use and indoor environments. It can simulate a building’s energy use throughout a typical year under varying meteorological conditions, including heating, cooling, lighting, and ventilation, and, by simulating indoor temperature, humidity, and airflow distribution, it assesses thermal comfort and provides data support for improving indoor environments.

Based on the space-state method, DeST establishes a physical model for simulating building thermal processes. Buildings are subject to both external and internal disturbances simultaneously: external disturbances primarily consist of environmental factors such as solar radiation and outdoor temperature, while internal disturbances mainly stem from occupants and equipment inside the building.

**Fig 29:**
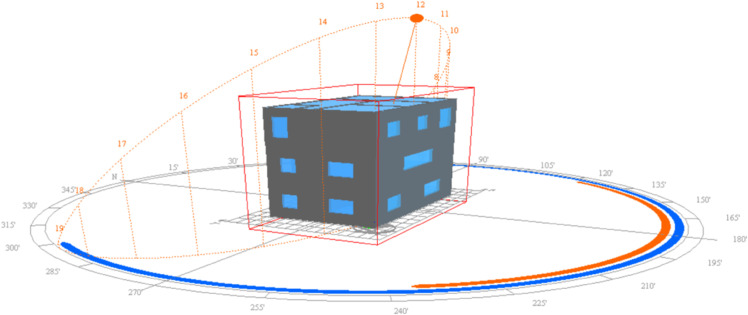
3D Model diagram.

The model is a three-story building with a height of 3.6 meters per floor, a length of 11.5 meters, and a width of 7.5 meters. The building’s form coefficient is 0.57, with a south-facing window-to-wall ratio of 0.14, a north-facing window-to-wall ratio of 0.06, a west-facing window-to-wall ratio of 0.11, and an east-facing window-to-wall ratio of 0.10, as shown in Fig 29. The selection of basic parameters for external walls, floors, windows, roofs, etc., is shown in Tables 8, 9, 10, and 11.

**Table 8:** Parameters of exterior wall materials.

Operating Condition	Material	Thickness (mm)	Dry Density (kg/m^3^)	Thermal Conductivity (W/(m⋅K))	Specific Heat Capacity (kJ/(kg⋅K))	Overall Thermal Resistance (W/K)	Total Heat Transfer Coefficient (W/(m^2^·K))
Condition A	Cement Mortar	20	1800	0.93	0.84	0.793	1.052
	Expanded Polystyrene Board	20	19	0.046	1.465		
	Cement Mortar	20	1800	0.93	0.84		
	Concrete Hollow Brick	240	1450	0.74	0.67		

**Table 9:** Floor material parameter table.

Type	Material	Thickness (mm)	Dry Density (kg/m^3^)	Thermal Conductivity (W/(m⋅K))	Specific Heat Capacity (kJ/(kg⋅K))
Floor	Reinforced Concrete	120	1000	1.00	0.88
	Rigid Polyurethane Board	20	30	0.03	1.38
	Reinforced Concrete	40	1000	1.00	0.88

**Table 10:** Exterior window parameter table.

Type	Thermal Conductivity *K* (W/m^2^·K)	Solar Heat Gain Coefficient	Frame Material
Exterior Window	1.0	0.35	Thermal Break Aluminum

**Table 11:** Roof material parameter table.

Type	Material	Thickness (mm)	Dry Density (kg/m^3^)	Thermal Conductivity (W/(m⋅K))	Specific Heat Capacity (J/(kg⋅K))
Roof	Rigid Foam Insulation	40	2500	1.63	837
	Reinforced Concrete	200	37	0.033	0.73
	Asphalt Shingle	20	1800	0.93	1050

### 5.1 Analysis of the impact of thicknesses of insulation materials on building energy consumption

**Fig 30:**
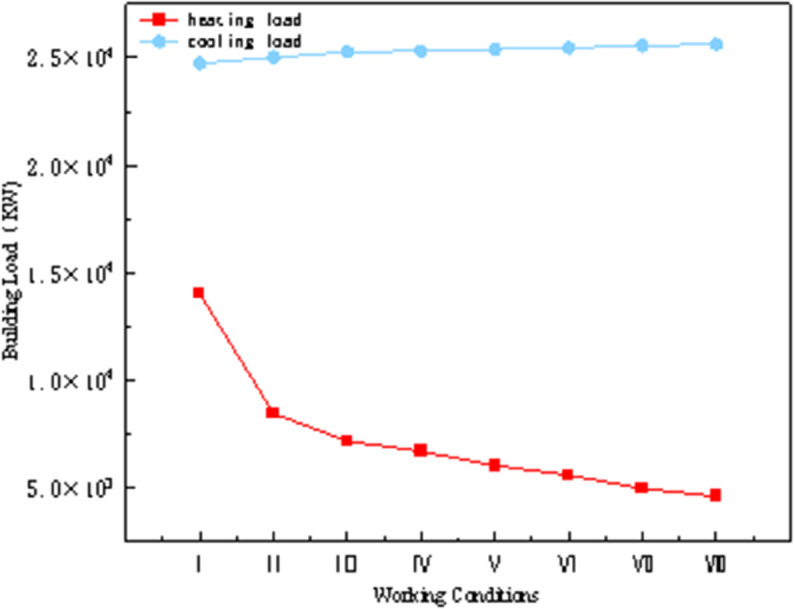
Annual cooling and heating load of building (different conditions).

**Fig 31:**
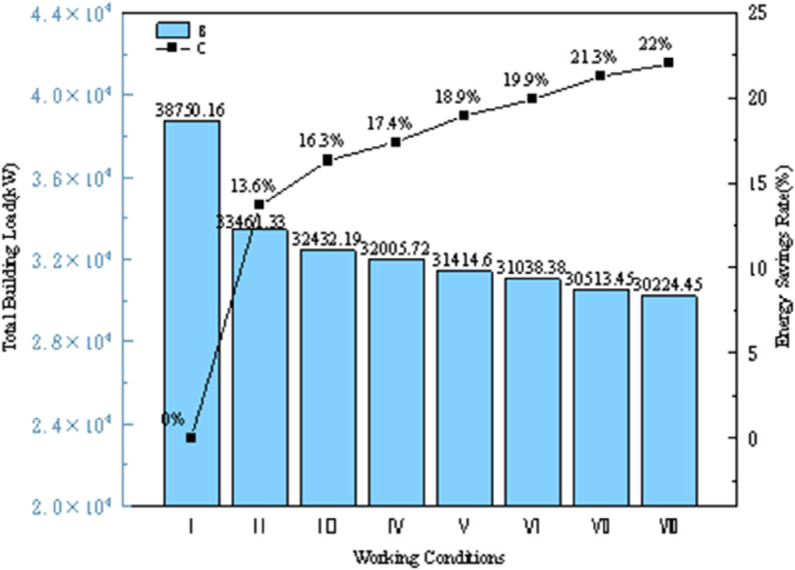
Total building load and energy saving rate (different conditions).

As shown in Table 12, the external wall insulation material is selected as GXPS board, with insulation thicknesses of 40, 50, 60, 80, 100, 150, and 200 mm. Weather parameters are imported using typical year data for Hefei City to simulate the annual energy consumption of the model. The annual cooling and heating loads are illustrated in Fig 30, and the energy-saving rates for each condition are analyzed as shown in Fig 31.

**Table 12:** Different thicknesses of insulation materials: Wall simulation conditions.

Condition	Material	Thickness (mm)	Dry Density (kg/m^3^)	Thermal Conductivity (W/(m⋅K))	Specific Heat Capacity (kJ/(kg⋅K))	Overall Heat Transfer Coefficient (W/(m^2^·K))
II	GXPS Board	40	30	0.024	0.732	0.417
	Aerated Concrete Block	100	566	0.174	1.047	
III	GXPS Board	50	30	0.024	0.732	0.355
	Aerated Concrete Block	100	566	0.174	1.047	
IV	GXPS Board	60	30	0.024	0.732	0.309
	Aerated Concrete Block	100	566	0.174	1.047	
V	GXPS Board	80	30	0.024	0.732	0.246
	Aerated Concrete Block	100	566	0.174	1.047	
VI	GXPS Board	100	30	0.024	0.732	0.204
	Aerated Concrete Block	100	566	0.174	1.047	
VII	GXPS Board	150	30	0.024	0.732	0.143
	Aerated Concrete Block	100	566	0.174	1.047	
VIII	GXPS Board	200	30	0.024	0.732	0.110
	Aerated Concrete Block	100	566	0.174	1.047	

In the simulation, the thickness of the insulation material is varied to alter the overall heat transfer coefficient of the walls. Fig 30 presents the total building loads and energy savings for walls with different heat transfer coefficients. In Condition I, the insulation material is traditional expanded polystyrene, which serves as the baseline for calculating energy savings. It is evident that using GXPS boards significantly improves insulation performance. The energy-saving rate increases with the heat transfer coefficient, ranging from 13.6% to 22.0% for Conditions II to VIII. Condition III saves 2.7% more than Condition II, while Condition VIII saves an additional 0.8% compared to Condition VII. The rate of increase in energy savings gradually diminishes, with the 50 mm thickness showing the most pronounced advantage in energy savings.

As shown in Fig 31, the heating load decreases with increasing heat transfer coefficients, while the cooling load gradually increases. Although the insulation performance improves, its thermal insulation needs to be balanced, but the increase in cooling load is much smaller than the decrease in heating load. Due to the climatic characteristics of central and southern Anhui, summers are hot, transitional seasons have suitable temperatures, and winters are cold. Additionally, as the thickness of the insulation layer increases, both the unit cost and construction difficulty also rise. Therefore, based on the trends illustrated, the 50 mm thick GXPS board insulation material is more suitable for this region.

To further investigate the impact of different insulation thicknesses on building energy consumption in central and southern Anhui, climate parameters from Huangshan City were selected, and simulations were conducted using thicknesses of 50, 100, and 200 mm. This analysis draws on common data from Hefei and Huangshan.

**Fig 32:**
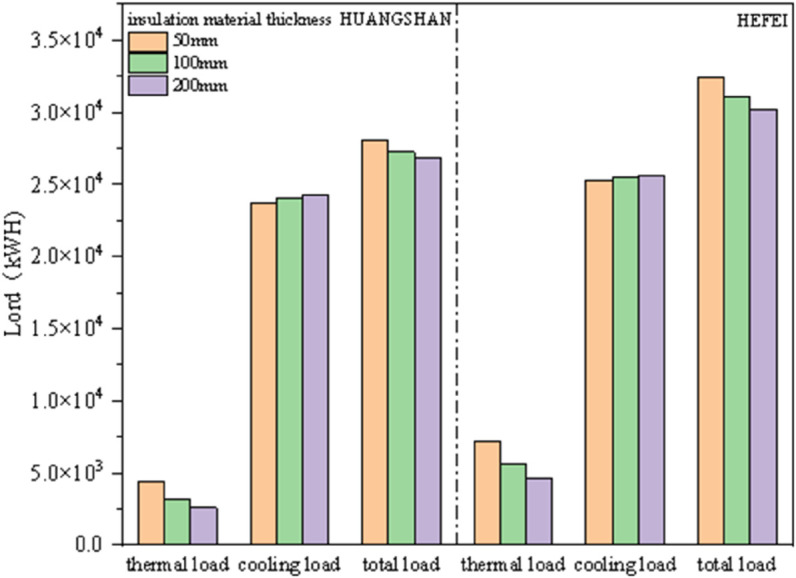
Impact of insulation thickness on annual building load).

As shown in Fig 32, the cooling load in Hefei and Huangshan is significantly higher than the heating load, with Huangshan’s annual load overall being lower than that of Hefei. It is also evident that as the thickness of the insulation material increases, the heating load gradually decreases, while the cooling load shows a slight increase. The total load exhibits a downward trend, particularly with a noticeable decrease from 50 mm to 100 mm, while the change from 100 mm to 200 mm is less pronounced.

This indicates a consistent overall trend between Hefei and Huangshan: increasing the thickness of the insulation material raises construction difficulty and, from an economic perspective, increases costs. Therefore, considering the energy-saving requirements, a thickness of 50 mm is deemed suitable.

From an economic perspective, although the initial cost of the GXPS board (approximately 900 RMB/m^3^) is about three times that of ordinary expanded polystyrene (EPS) board (approximately 300 RMB/m^3^), a configuration of 50 mm GXPS combined with 100 mm aerated concrete can achieve an excellent exterior wall heat transfer coefficient of 0.355 W/(m^2^⋅K) and deliver 16.3% energy savings compared with conventional practices. In addition, its distinctive thermal response characteristics—rapid heat retention in winter and slower heat conduction in summer—are well aligned with the local climate featuring cold winters and hot summers. Considering life-cycle costs, GXPS can significantly reduce building operating energy use and avoid future retrofit risks, thereby offering superior overall economic performance. It is an ideal choice for meeting the 75% energy-saving standard and the requirements of nearly zero energy buildings. By contrast, EPS is suitable only for projects with tight budgets or temporary buildings with low energy-saving requirements; over the long term, it risks insufficient energy performance and potential retrofit needs.

## 6 Conclusion

This paper conducted a preliminary exploration of the application and performance optimization of nearly zero energy building materials in central and southern Anhui, addressing several key technical issues and achieving certain research results.

(1) Through the analysis of the thermal performance of opaque building envelopes, the study tested and researched the performance of two new types of insulation materials available in the market (GXPS board and SEPS board) under different temperature and humidity conditions. The experimental results show that: GXPS exhibits excellent thermal insulation performance during transitional seasons and winter, with a thermal conductivity as low as 0.023 W/(m⋅K), an average temperature difference between internal and external surfaces *leq*6.5°C, and stable heat flux density fluctuations under extreme temperature differences (30°C). SEPS performs exceptionally in humid and hot summer environments, with significantly uniform temperature distribution, and its thermal inertia characteristics can suppress summer daytime heat penetration, reducing air conditioning dehumidification loads, making it suitable for high-temperature and high-humidity climate zones. Both materials demonstrate excellent thermal insulation performance in building roofs, floors, and external walls, capable of meeting the 75% energy-saving requirements of most cities in China, as well as the insulation needs of passive ultra-low energy buildings and nearly zero energy buildings.

(2) GXPS responds rapidly during cooling processes, matching the rapid cooling climate characteristics of winter; SEPS’s heating hysteresis can mitigate the impact of high-temperature shocks during summer, and the two materials, through complementary thermal characteristics (low thermal conductivity and high humidity stability), can effectively address the climate in central and southern Anhui.

(3) Using GXPS board as a wall insulation material, building energy consumption simulations were conducted by setting up eight different thickness conditions from 40 mm to 200 mm. The research shows that: as the insulation material thickness increases, the total building load and heating load significantly decrease, while the annual cooling load slightly increases, with the energy-saving rate rising from 13.6% at 40 mm to 22.0% at 200 mm. After comprehensive evaluation, the 50 mm thick GXPS board performs best when applied in central and southern Anhui.

(4) With GXPS exhibiting low thermal conductivity and fast cooling response in winter and during transitional seasons, and SEPS showing uniform temperature distribution and pronounced thermal inertia under hot and humid summer conditions, their complementary thermal characteristics suggest future optimization of material placement tailored to the “hot summer and cold winter” climate of central and southern Anhui. Exterior walls can be differentiated by orientation, using SEPS on the south to suppress summer heat ingress and 50 mm GXPS on the north to reduce winter heating demand; roofs and floors can adopt a composite design with “SEPS as the outer layer + GXPS as the inner layer”. Further studies can be conducted on long-term hygrothermal aging of both materials, optimization of thermal bridges at junctions, and coordinated application with renewable energy systems. Research can also be extended to the recycling and regeneration pathways of these graphite-modified materials to reduce carbon emissions during production, thereby providing more precise technical support for the envelope design of nearly zero-energy buildings in this region.
